# Effect of fecal microbiota transplantation in patients with slow transit constipation and the relative mechanisms based on the protein digestion and absorption pathway

**DOI:** 10.1186/s12967-021-03152-2

**Published:** 2021-12-01

**Authors:** Lulu Xie, Chen Xu, Yadong Fan, Yuwei Li, Ying Wang, Xiaoyu Zhang, Shuang Yu, Jida Wang, Rundong Chai, Zeyu Zhao, Yutong Jin, Zhe Xu, Shuwu Zhao, Yuhong Bian

**Affiliations:** 1 School of Medicine, Nankai University, Tianjin, 300071 China; 2 Department of Colorectal Surgery, Tianjin Union Medical Center, Tianjin, 300121 China; 3 School of Intergrative Medicine, Tianjin University of Traditional Chinese Medicine, 10 Poyanghu Road, Tianjin, 301617 China; 4 National Clinical Research Center for Chinese Medicine Acupuncture and Moxibustion, First Teaching Hospital of Tianjin University of Traditional Chinese Medicine, Tianjin, 300193 China

**Keywords:** Fecal microbiota transplantation (FMT), Slow transit constipation (STC), The protein digestion and absorption pathway

## Abstract

**Background:**

Fecal microbiota transplantation (FMT) is considered an effective treatment for slow transit constipation (STC); nevertheless, the mechanism remains unclear.

**Methods:**

In this study, eight patients with STC were selected according to the inclusion and exclusion criteria; they then received three treatments of FMT. The feces and serum of STC patients were collected after each treatment and analyzed by integrating 16 s rRNA microbiome and metabolomic analyses.

**Results:**

The results showed that the percentage of clinical improvement reached 62.5% and the rates of patients’ clinical remission achieved 75% after the third treatment. At the same time, FMT improved the Wexner constipation scale (WCS), the Gastrointestinal Quality-of-Life Index (GIQLI) and Hamilton Depression Scale (HAMD). Fecal microbiome alpha diversity and beta diversity altered significantly after FMT. Analysis of the 16 s rRNA microbiome showed that the numbers of Bacteroidetes (Prevotell/Bacteroides) and Firmicute (Roseburia/Blautia) decreased, whereas Actinobacteria (Bifidobacterium), Proteobacteria (Escherichia), and Firmicute (Lactobacillus) increased after FMT. The metabolomics analyses showed that the stool of FMT-treated patients were characterized by relatively high levels of N-Acetyl-L-glutamate, gamma-L-glutamyl-L-glutamic acid, Glycerophosphocholine, et al., after FMT. Compared with baseline, the serum of treated patients was characterized by relatively high levels of L-Arginine, L-Threonine, Ser-Arg, Indoleacrylic acid, Phe-Tyr, 5-L-Glutamyl-L-alanine, and lower levels of Erucamide after the treatment. The correlation analysis between the metabolites and gut microbiota showed a significant correlation. For example, L-Arginine was positively correlated with lactobacillus, et al. L-Threonine was positively correlated with Anaerovibrio, Sediminibacterium but negatively correlated with Phascolarctobacterium. Erucamide had significant negative correlations with Sediminibacterium and Sharpea, while being positively correlated with Phascolarctobacterium. Enriched KEGG pathways analysis demonstrated that the protein digestion and absorption pathways gradually upregulated with the increase of FMT frequency. The L-Arginine and L-Threonine were also involved in the pathway. A large amount of Na + was absorbed in the pathway, so that it might increase mucus secretion and electrical excitability of GI smooth muscle.

**Conclusions:**

Therefore, we speculated that FMT changed the patients’ gut microbiota and metabolites involved in the protein digestion and absorption pathways, thereby improving the symptoms of STC. Study on the effectiveness and safety of FMT in the treatment of STC. The study was reviewed and approved by Ethics Committee of Tianjin People's Hospital (ChiCTR2000033227) in 2020.

## Introduction

Chronic constipation is a prevalent gastrointestinal disorder, with pooled prevalence of 14% across the world [1]. With changes of lifestyle, the incidence of chronic constipation continues to increase year by year. Chronic constipation can be divided into slow transit constipation (STC), defecatory disorder, normal transit constipation (NTC) and mixed constipation according to the pathophysiological mechanism [2]. STC is a common type of chronic constipation characterized by the delayed emptying of the ascending and transverse colon and extended gut transit time as measured by radiopaque markers [3]. It is usually encountered in middle-aged women, seriously affecting their quality of life and posing a considerable economic burden. Research shows that the Psychological General Well­Being Index (PGWBI) and the patient assessment of constipation QOL (PAC-QOL) scores were worse in patients suffering constipation compared with individuals with no constipation, on the basis of the Rome criteria [4]. The STC patients also usually experienced mood disorders, such as depression, anxiety and somatoform-type behaviors [5–7]. At the same time, patients also suffered associated gastrointestinal tract and abdominal symptoms, such as dyspepsia, Irritable Bowel Syndrome (IBS) and bloating [8]. However, the pathogenesis of STC remains unclear. Lifestyle modifications, pharmacological therapy and surgical intervention are important management strategies for the treatment of STC, such as increasing dietary fiber, physical activity, osmotic laxatives, lubiprostone, serotonergic agonists, anorectal biofeedback therapy, et al. Unfortunately, current treatments are not satisfactory in half the constipated individuals because of the lack of efficacy and having many adverse effects [9]. There is little support concerning the effectiveness of lifestyle modification and osmotic laxatives [10, 11]. Moreover, many common adverse effects of pharmacological therapy such as diarrhea, nausea, abdominal pain and headache have been reported [12]. Therefore, it is necessary to find new treatments to address these issues and improve patients’ quality of life.

Gut microbiota are emerging as important players in the human body such as the metabolic, neurological, and gut immune system. An increasing number of studies show that gastrointestinal microbiota are closely related to gut motility and constipation. A recent meta-analysis proved that microbiota decreased colonic transit time by 12.4 h, increased the frequency of bowel movements, and improved constipation-related symptoms [13]. Fecal microbiota transplantation (FMT) is proposed as a “stool transplant” from a healthy individual into the recipient. The goal of FMT is to normalize the composition of gut microbiota and treat the disease, such as clostridium difficile infection, metabolic syndrome, irritable bowel syndrome, et al. [14–16]. At present, FMT appears to be an effective and safe therapeutic modality, with few adverse effects and has been successfully applied to STC [17].

However, the mechanism of gut microbiota for treating STC is unclear and remains to be explored. In this study, feces and serum were collected from STC patients before and after receiving FMT, and analyzed by integrating 16 s rRNA microbiome and metabolomic analyses. The aim of this research was to investigate the potential mechanism which may participate in the treatment of FMT.

## Materials and methods

### Patients criteria

#### Inclusion criteria

The diagnostic criteria of STC was based on the Rome IV criteria and colonic transit test. Rome IV criteria of constipation include: (1) The presence, in the preceding 6 months, of at least two of the following: (i) straining during more than one-fourth (25%) of defecations, (ii) lumpy or hard stools in more than one-fourth (25%) of defecations, (iii) sensation of incomplete evacuation in more than one-fourth (25%) of defecations, (iv) sensation of anorectal obstruction/blockage in more than one-fourth (25%) of defecations, (v) manual maneuvers to facilitate in more than one fourth (25%) of defecations (e.g., digital evacuation, support of the pelvic floor), (vi) fewer than three spontaneous bowel movements per week. (2) No loose stool without the use of laxative. (3) Inadequate evidence for the diagnosis of irritable bowel syndrome.

All patients underwent colonoscopy to exclude any luminal pathology or secondary causes of constipation. Patients were dissatisfied with traditional treatment (including dietary modification, laxatives, biofeedback, et al.) for more than six months. STC patients were diagnosed by colonic transit test and colonic transit time > 48 h. Other criteria included age ≥ 18 years and body mass index (BMI): 18.5–25 kg/m^2^.

#### Exclusion criteria

Exclusion criteria included:

(1) Congenital megacolon; (2) constipation caused by secondary intervention (e.g., drugs, metabolic disorder, endocrine disorders or neurological disorders); (3) previous abdominal surgery (except for cholecystectomy, appendectomy, cesarean and tubal ligation) and perianal surgery; (4) history of or current gastrointestinal disease (e.g., malignancy, IBD); (5) infected with enteric pathogens; (6) used prebiotics, probiotics or proton pump inhibitors during the past month; (7) pregnant or breast-feeding women; (8) long-term smoking and/or alcohol addiction; (9) confirmed to have hepatic, renal, cardiovascular, respiratory or psychiatric disease; (10) suffered from other disease which could affect intestinal transit and gut microbiota.

### Donor screening

(1) Volunteers were healthy adults, without any other disease especially digestive system diseases, and excepting pregnant woman (BMI: 18.5–25 kg/m^2^). (2) Eligible donors had not received antibiotics, probiotics, proton pump inhibitors and other drugs that affect intestinal microecology, for at least six months before FMT donation. (3) The results of blood screening tests were negative for hepatitis A, B and C, HIV, syphilis, Treponema Pallidum, et al. (4) Stool screening tests were negative for Clostridium Difficile, Shigella, Yersinia, Campylobacter, parasites and other common enteric pathogens.

### Preparation of fecal suspension

Approximately 100 g of fresh stool was immediately homogenized in a blender with 500 mL of 0.9% sterile saline for several seconds. Afterwards, it was passed through a decreasing number of stainless steel sieves (2.0 to 1.0 to 0.5 to 0.25 mm), several times, to remove the small particles [18]. Then, sterile glycerol was amended in the filtered suspension with a final concentration of 10%. The supernatant was stored at -80℃ or administered to the patients immediately.

### FMT procedures

Eligible patients were given the antibiotics orally (vancomycin 500 mg two times per day) for 3 consecutive days. Then, a nasojejunal tube was placed in the patient's proximal jejunum under endoscopy. The patients stopped other conventional treatments on constipation for three days and did not eat anything for eight hours before FMT. Sodium phosphate (90 mL) was given to empty the bowel after the antibiotic treatment. After 12 h of rest, 50 mL fecal suspension was infused slowly into patients through the nasojejunal tube within 5 min. Then 15 mL saline solution was used to flush the nasojejunal tube. The procedure was performed for six consecutive days, once every half month and repeated three times. The patient's stool and serum samples were collected the day before FMT (Baseline-B1) and after per-FMT (PostFMT-B2, B3, B4). The patients were reminded to record symptom severity, stool consistency and maintain healthy habits during the treatment of FMT. Antibiotics were not allowed during the period. If the patients did not have a bowel movement for three or more than three days, they were permitted to use 20 g of Macrogol 4000 powder (Forlax®, Ipsen, France). If ineffective, patients were allowed to use enema. Use of rescue medication was recorded.

### Outcome measures

#### Primary outcome measure

(1) Clinical remission rate: the proportion of patients who had, on average, three or more spontaneous complete bowel movements (SCBMs) per week. (2) Clinical improvement rate: the proportion of patients whose clinical symptoms had not completely disappeared, but significantly improved when compared with the baseline or patients with an average increase of one or more SCBMs/week. (3) Safety assessments: adverse events which contained any gastrointestinal symptoms such as abdominal pain, nausea, flatulence, et al., were recorded immediately during the FMT treatment and follow-up period.

#### Secondary outcome measure

(1) WCS, an internationally adopted questionnaire, which is used to quantify the severity of constipation [19]. This consists of a 30-point and 8-point questionnaire to examine the various clinical symptoms of constipation, with scores ranging from 0 (best) to 30 (worst). According to the scale, the score of healthy people is < 8 points; anything over is considered to be constipation [20]. (2) The GIQLI is a well-validated questionnaire to evaluate patients’ gastrointestinal symptoms and specific quality of life, such as psychological. It comprises 36 questions using a five-point Likert-type scale (0, worst; 4, best). The mean scores in healthy subjects were 125.8. (3) HAMD is an evaluation index to assess depression symptoms. Patients were considered to be suffering from depression with scores of > 7.

### Statistical analysis

All data were analyzed using SPSS Version 20.0 software. Continuous data were presented as mean ± SD (M ± SD), whereas categorical data were presented as n (%). Student t-test was performed to analyze normally distributed continuous variables, and χ^2^ test was used for categorical variables as appropriate. R value was used to evaluate the degree of correlation. P < 0.05 was considered to be statistically significant.

### 16 s rRNA microbiome and metabolomic analyses

#### Extraction of genome DNA

Total genome DNA from samples was extracted using the CTAB/SDS method. DNA concentration and purity was monitored on 1% agarose gels. According to the concentration, DNA was diluted to 1 ng/μL using sterile water.

#### 16S rRNA gene PCR and amplicon sequencing

Primer: 16S V4: 515F-806R. 16S rRNA genes were amplified used the specific primer with the barcode. All PCR reactions were carried out in 30 μL reactions with 15 μL of Phusion® High-Fidelity PCR Master Mix (New England Biolabs); 0.2 μM of forward and reverse primers, and about 10 ng template DNA. Thermal cycling consisted of initial denaturation at 98 ℃ for 1 min, followed by 30 cycles of denaturation at 98 ℃ for 10 s, annealing at 50 ℃ for 30 s, and elongation at 72 ℃ for 30 s. Finally, 72 ℃ for 5 min. The same volume of 1X loading buffer (contained SYB green) with PCR products was mixed and electrophoresis operated on 2% agarose gel for detection. Samples with a bright main strip between 400 and 450 bp were chosen for further experiments. PCR products were mixed in equidensity ratios. Then, the mixture of PCR products was purified with a GeneJET Gel Extraction Kit (Thermo Scientific). Sequencing libraries were generated using the TruSeq® DNA PCR-Free Sample Preparation Kit following the manufacturer’s recommendations and index codes were added. The library quality was assessed on the Qubit@ 2.0 Fluorometer (Thermo Scientific) and Agilent Bioanalyzer 2100 system. Finally, the library was sequenced on an Illumina HiSeq 2500 and 250 bp paired-end reads were generated.

#### Metabolite extraction and UPLC-Q-TOF–MS detection

LC–MS/MS analysis (HILIC/MS) was performed using a UHPLC (1290 Infinity LC, Agilent Technologies) coupled to a quadrupole time-of-flight (AB Sciex TripleTOF 6600). For HILIC separation, samples were analyzed using a 2.1 mm × 100 mm ACQUIY UPLC BEH 1.7 µm column (Waters, Ireland). In both ESI positive and negative modes, the mobile phase contained A = 25 mM ammonium acetate and 25 mM ammonium hydroxide in water and B = acetonitrile. The gradient was 85% B for 1 min and was linearly reduced to 65% in 11 min, and then reduced to 40% in 0.1 min and kept for 4 min, and then increased to 85% in 0.1 min, with a 5 min re-equilibration period employed. The ESI source conditions were set as follows: Ion Source Gas1 (Gas1) as 60, Ion Source Gas2 (Gas2) as 60, curtain gas (CUR) as 30, source temperature: 600℃, IonSpray Voltage Floating (ISVF) ± 5500 V. In MS-only acquisition, the instrument was set to acquire over the m/z range 60–1000 Da, and the accumulation time for TOF MS scan was set at 0.20 s/spectra. In auto MS/MS acquisition, the instrument was set to acquire over the m/z range 25–1000 Da, and the accumulation time for product ion scan was set at 0.05 s/spectra. The product ion scan is acquired using information dependent acquisition (IDA) with high sensitivity mode selected. The collision energy (CE) was fixed at 35 V with ± 15 eV. Declustering potential (DP) was set as ± 60 V. LC–MS/MS analysis (RPLC/MS) was performed using a UHPLC (1290 Infinity LC, Agilent Technologies) coupled to a quadrupole time-of-flight (AB Sciex TripleTOF 6600). For RPLC separation, a 2.1 mm × 100 mm ACQUIY UPLC HSS T3 1.8 µm column (Waters, Ireland) was used. In ESI positive mode, the mobile phase contained A = water with 0.1% formic acid and B = acetonitrile with 0.1% formic acid; and in ESI negative mode, the mobile phase contained A = 0.5 mM ammonium fluoride in water and B = acetonitrile. The gradient was 1% B for 1.5 min and was linearly increased to 99% in 11.5 min and kept for 3.5 min. Then, it was reduced to 1% in 0.1 min and 3.4 min re-equilibration period was employed. The gradients were at a flow rate of 0.3 mL/min, and the column temperatures were kept constant at 25℃. A 2 µL aliquot of each sample was injected. The ESI source conditions were set as follows: Ion Source Gas1 (Gas1) as 40, Ion Source Gas2 (Gas2) as 80, curtain gas (CUR) as 30, source temperature: 650℃, IonSpray Voltage Floating (ISVF) 5000 V in positive mode, and − 4000 V in negative mode. In MS-only acquisition, the instrument was set to acquire over the m/z range 60–1000 Da, and the accumulation time for TOF MS scan was set at 0.20 s/spectra. In auto MS/MS acquisition, the instrument was set to acquire over the m/z range 25–1000 Da, and the accumulation time for product ion scan was set at 0.05 s/spectra. The product ion scan was acquired using information dependent acquisition (IDA) with high sensitivity mode selected. The collision energy (CE) was fixed at 35 V with ± 15 eV. Declustering potential (DP) was set as ± 60 V.

#### Data analysis

Paired-end reads from the original DNA fragments were merged using FLASH [21]. Paired-end reads were assigned to each sample according to the unique barcodes. Sequences were analyzed using the QIIME [22] software package (Quantitative Insights into Microbial Ecology), and in-house Perl scripts were used to analyze alpha- (within samples) and beta- (among samples) diversity. First, reads were filtered by QIIME quality filters. Then, operational taxonomic units (OTUs) were picked by making an OTU table. Sequences with ≥ 97% similarity were assigned to the same OTUs. A representative sequence for each OTU was selected and annotated with taxonomic information by the RDP classifier [23]. In order to compute Alpha Divesity, the OTU table was rarified and calculated by Shannon index. Rarefaction curves were generated based on these three metrics. QIIME calculates both weighted and unweighted unifrac, which are phylogenetic measures of beta diversity. Unweighted unifrac for Principal Coordinate Analysis (PCoA) was used to obtain principal coordinates and visualize them from complex, multidimensional data.

The raw MS data (wiff.scan files) were converted to MzXML files using ProteoWizard MSConvert and processed using XCMS for feature detection, retention time correction and alignment. The metabolites were identified by accuracy mass (< 25 ppm) and MS/MS data which were matched with our standards database. In the extracted ion features, only the variables having more than 50% of the nonzero measurement values in at least one group were kept. For the multivariate statistical analysis, the MetaboAnalyst (www.metaboanalyst.ca) web-based system was used. After Pareto scaling, principal component analysis (PCA) and partial least-squares-discriminant analysis (PLS-DA) were performed. The leave one out cross-validation and response permutation testing was used to evaluate the robustness of the model. The significant different metabolites were determined based on the combination of a statistically significant threshold of variable influence on projection (VIP) values obtained from PLS-DA model and two-tailed Student’s t test (p value) on the raw data, and the metabolites with VIP values larger than 1.0 and p values less than 0.1 were considered as significant.

## Results

### Patients’ characteristics

The baseline demographic features and clinical characteristics of the patients with STC who received FMT are summarized in Table 1. According to the inclusion and exclusion criteria, eight patients (two males and six females) were included in this study. The age of patients was 43.75 ± 16.95 (y), weight was 54.12 ± 7.35 (kg), BMI was 20.10 ± 2.28 (kg/m^2^), disease duration was 9.50 ± 8.99 (y) and CSBMs was 1.5 ± 0.53 per week. All patients received three FMT treatments during the study. Stool and serum samples were collected before FMT(marked as B1) and after each treatment(marked as B2, B3, B4). Not all stool and serum samples were collected after each FMT. So, ultimately, four patients had B1, B2, B3, B4; one patient had B1, B2, B3; two patients had B1, B2; one patient had B1, B4.

**Table 1:** Characteristics of included patients

Patient no.	Sex	Age (y)	Weight (kg)	BMI (kg/m^2^)	Disease duration (y)	CSBMs, per week
1	M	61	67.5	23.08	5	2
2	F	35	50	19.29	10	1
3	F	27	50	19.53	2	1
4	F	60	46	17.3	4	2
5	F	35	47.5	17.66	22	2
6	F	34	57	22.83	25	2
7	F	25	54	19.83	3	1
8	M	73	61	20.62	5	1
Total (M ± SD)	–	43.75 ± 16.95	54.12 ± 7.35	20.10 ± 2.28	9.50 ± 8.99	1.5 ± 0.53

*F* female, *M* male, *BMI* body mass index, *CSBM* complete spontaneous bowel movements

### Effects on primary and secondary endpoints

The results show that symptoms of patients with STC were significantly improved after FMT compared with the baseline (Table 2). After treatment with FMT, the percentage of clinical improvement over the intervals of B2, B3 and B4 respectively reached to 62.5%, 50% and 62.5%. The rates of patients’ clinical remission were achieved in 87.5%, 62.5% and 75% over the intervals B2, B3 and B4, respectively. The WCS demonstrated a significant reduction from B1 to B4: B1 (12.12 ± 4.05), B2 (7.62 ± 3.85), B3 (6.87 ± 4.15) and B4 (7.12 ± 3.52), indicating the effectiveness of FMT (P < 0.05). The GIQLI scores were significantly increased compared with the baseline (P < 0.05) and represented the improvement in the patient’s quality of life. HAMD, which was used to assess the symptoms of depression, showed a clear downward trend after FMT treatment.

**Table 2:** Clinical outcomes in patients undergoing FMT

Project	B1	B2	B3	B4
Clinical improvement rate (%)	0	62.5% (5/8)	50% (4/8)	62.5% (5/8)
Clinical remission rate (%)	0	87.5% (7/8)	62.5% (5/8)	75% (6/8)
WCS	12.12 ± 4.05	7.62 ± 3.85^#^	6.87 ± 4.15^#^	7.12 ± 3.52^#^
GIQLI	89.12 ± 16.54	115.37 ± 17.18^#^	123.25 ± 12.04^#^	131.12 ± 6.22^#^
HAMD	7.75 ± 6.73	4.12 ± 2.69^#^	2.87 ± 2.29^#^	2.37 ± 2.06^#^

Data are expressed as the M ± SD

B1, before the treatment; B2, after the first treatment; B3, after second treatment; B4, after third treatment

^#^p < 0.05 and the difference was statistically significant compared with B1 (before the treatment) values

There were five patients who had nausea and three patients had flatulence when the nasointestinal tube was inserted, but those discomforts disappeared when the tube was removed. One patients was observed with occasional diarrhea and two patients reported borborygmi. These mild adverse events gradually reduced or disappeared. No serious adverse reactions occurred during the FMT period and the mild adverse events have been recorded.

### FMT-induced changes in fecal bacteria of patients

**Fig. 1:**
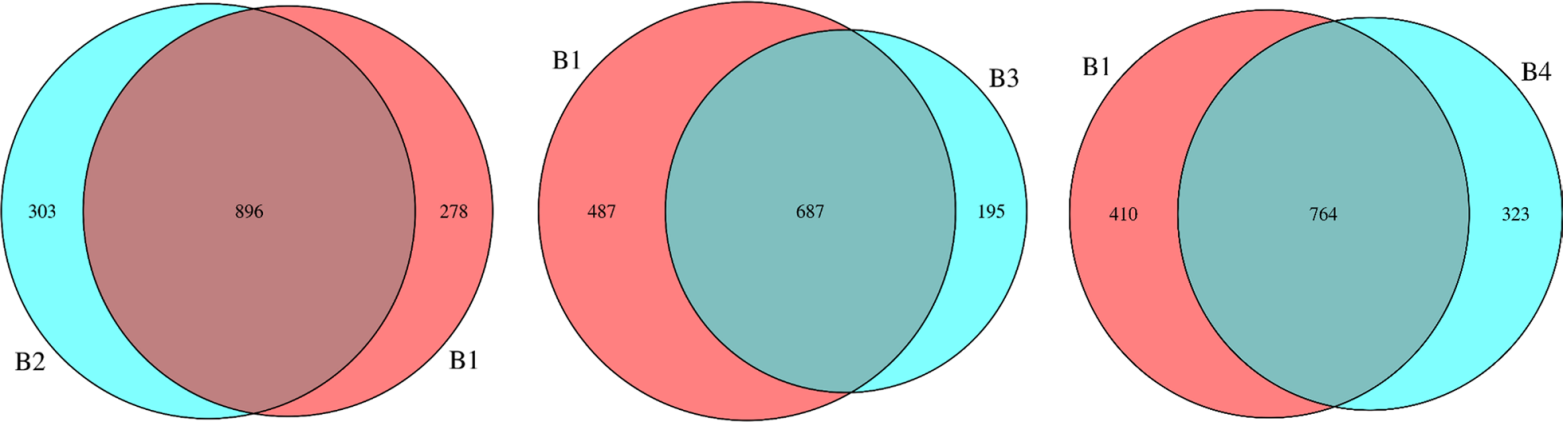
Venn diagram displayed the common and unique OTUs of gut microbiota between baseline and post-FMT. The size of the circle represents the number of OTUs. The larger the circle, the greater the number of OTUs. On the contrary, the less

**Fig. 2:**
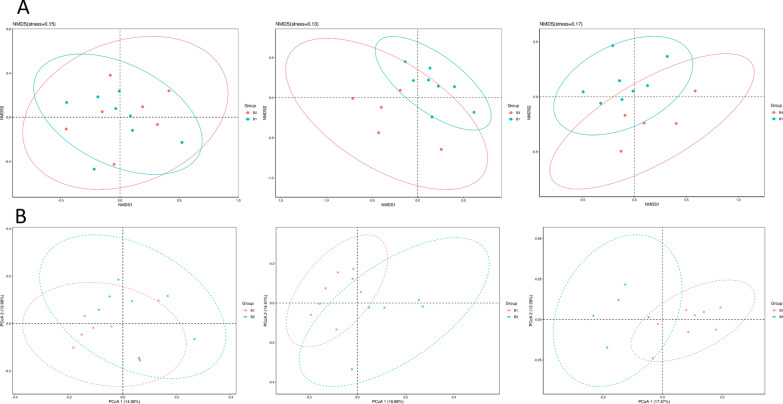
NMDS (**A**) and PCoA (**B**) of taxonomic abundances assessed by 16S rRNA gene sequencing confirm that post-FMT samples tend to cluster farther compared to the baseline samples

**Fig. 3:**
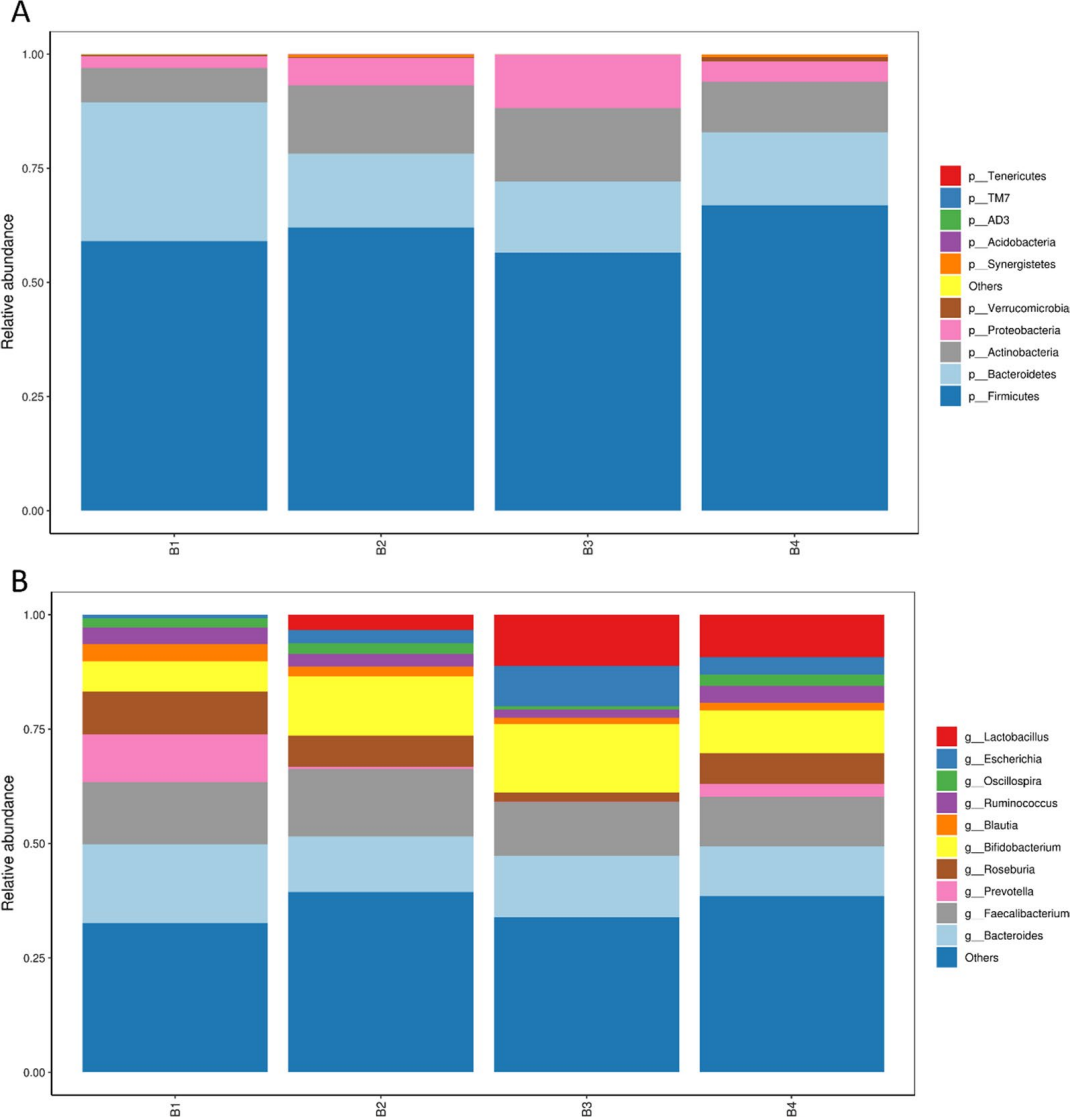
The phylum-level (**A**) and genus-level (**B**) abundances of fecal microbiota in FMT patients at baseline and post-FMT

**Fig. 4:**
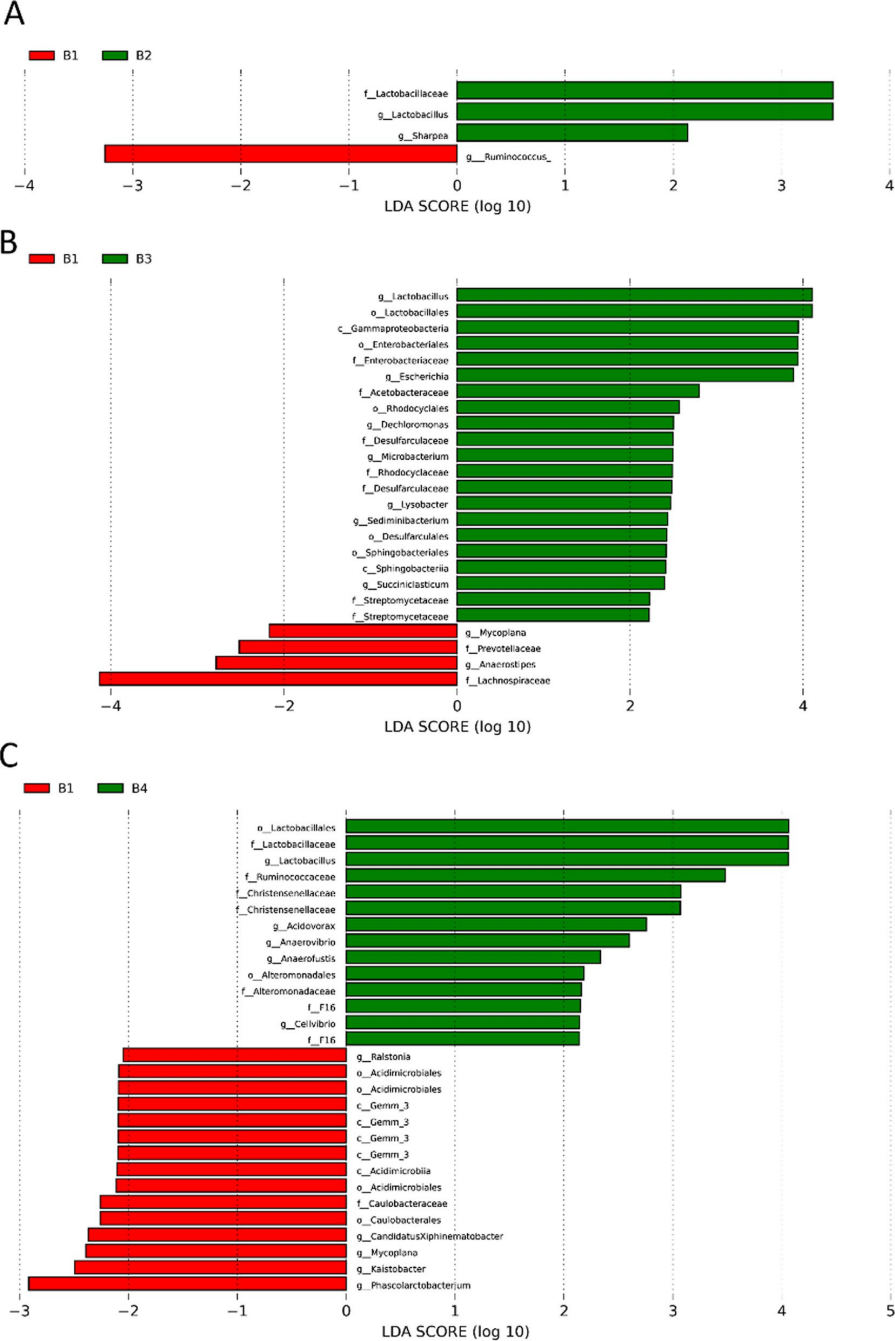
LEfSe analysis with an LDA value of > 2 displayed the dominant microbiomes in each group

The fecal microbiomes of these patients were analyzed by 16 s rRNA gene sequencing. Veen diagrams, nonmetric multidimensional scaling (NMDS), Principal Co-ordinates Analysis (PCoA), the histogram of species relative abundance, and LDA Effect Size (LEfSe) analysis were used to assess the species enrichment and distribution in each group. The composition of gut microbiota was changed significantly in all participants. Fecal alpha diversity (within the sample diversity), measured as a Venn diagram displayed the common and unique OTUs between baseline and post-FMT (Fig. 1). After the first FMT, there were 896 common OTUs and 581 unique OTUs in the baseline and the post FMT patients; after the second FMT, 687 common OTUs and 682 unique OTUs between the B1 and B3; after the third treatment, 764 common OTUs and 733 unique OTUs between the B1 and B4. We found that the number of unique OTUs gradually increased and the number of common OTUs generally showed a downward trend with continuous treatment. Microbiome beta diversity (between sample diversity), which was measured by NMDS and PCoA (Fig. 2) also altered significantly with FMT, suggesting that FMT transferred the microbial communities of the patients after treatment. The histogram of species relative abundance was used to show the top 10 species in the family-level and genus-level according to the OTU results. The composition of fecal microbiota in the post FMT patients significantly differed from baseline in the phylum-level and genus-level abundances (Fig. 3). The analysis showed that the number of Bacteroidetes (Prevotell/Bacteroides), Firmicute (Roseburia/Blautia) decreased, and Actinobacteria (Bifidobacterium), Proteobacteria (Escherichia), Firmicute (Lactobacillus) increased in general. The dominant microbiomes in each group were displayed by LEfSe analysis with an LDA value of > 2 indicating statistical significance between two groups (Fig. 4). We found that the order Lactobacillales, family Lactobacillaceae, genus Lactobacillus were higher in the gut microbiota of the post-FMT group compared with baseline. The number of Sharpea, Sediminibacterium, Cellvibrio, Anaerofustis, et al., increased; Phascolarctobacterium, et al., decreased at different stages.

### Metabolomic profiles are changed significantly after FMT

**Fig. 5:**
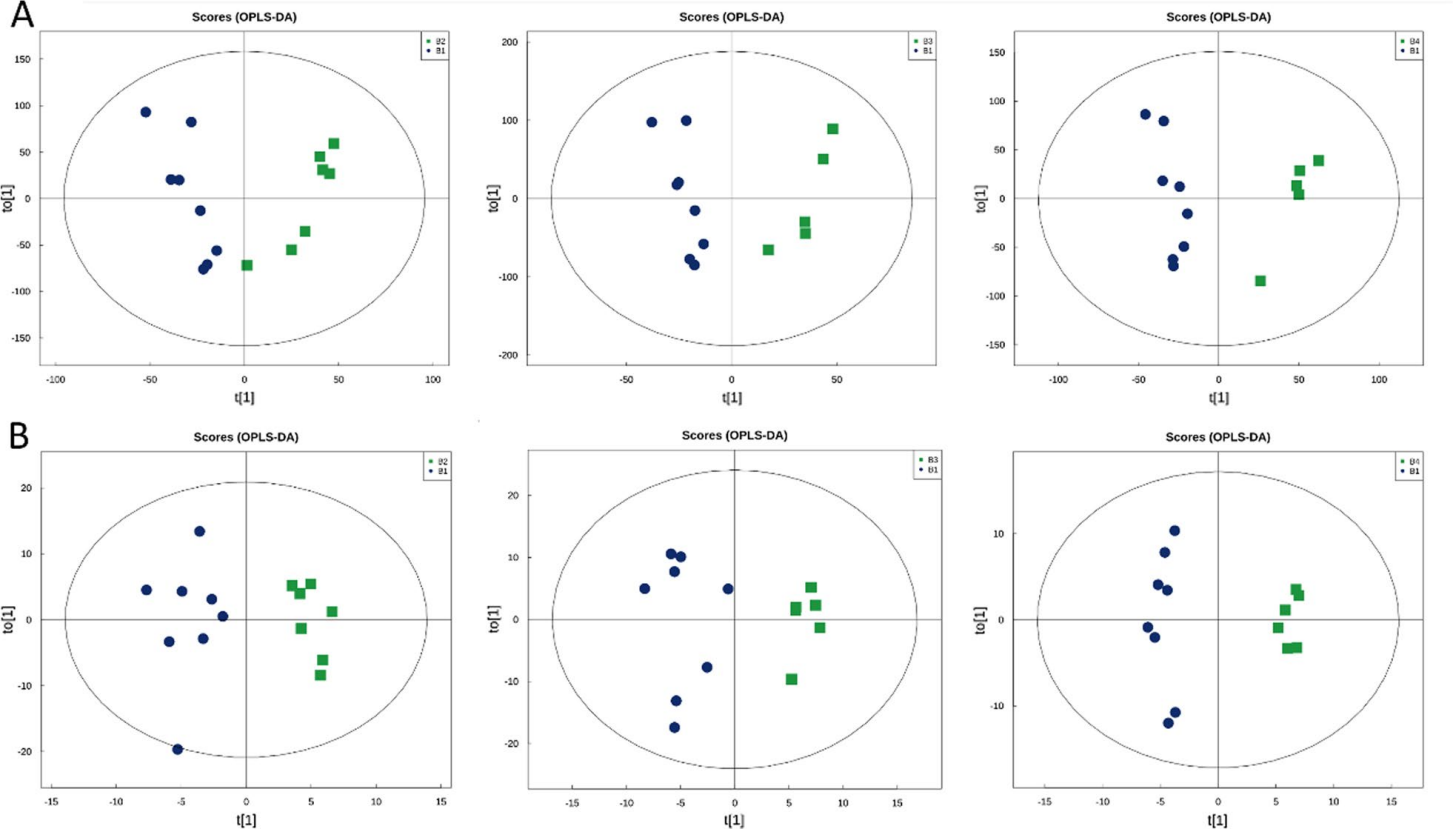
The OPLS-DA of feces (**A**) and serum (**B**) showed that the cluster of metabolites was significantly separated between baseline and post-FMT

**Fig. 6:**
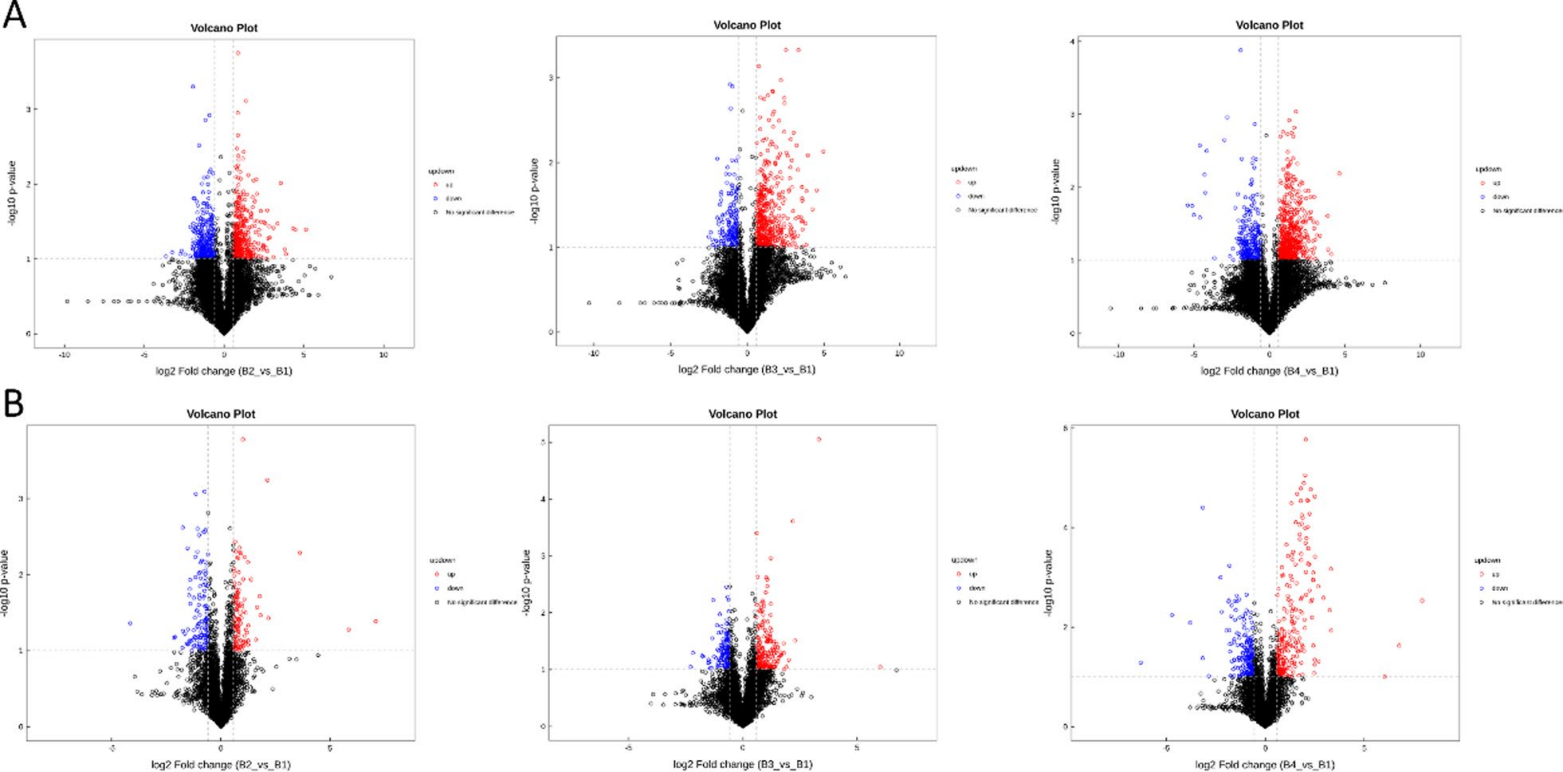
The univariate statistical analysis of Volcano plot showed the metabolite changes both in feces (**A**) and serum (**B**) between baseline and post-FMT

**Fig. 7:**
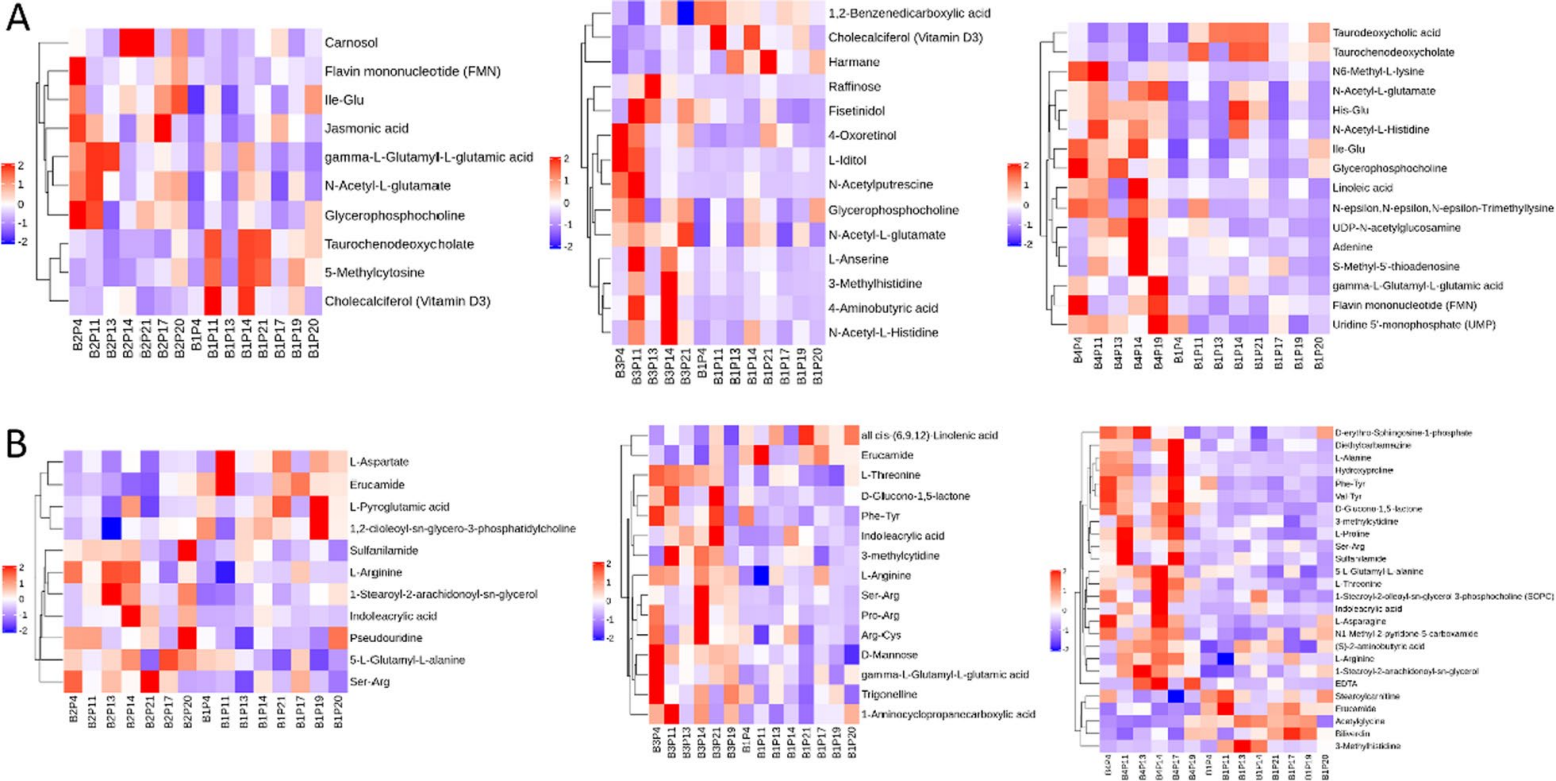
The univariate statistical analysis of Metabolites heatmap in showed the metabolite changes both in feces (**A**) and serum (**B**) between baseline and post-FMT

**Fig. 8:**
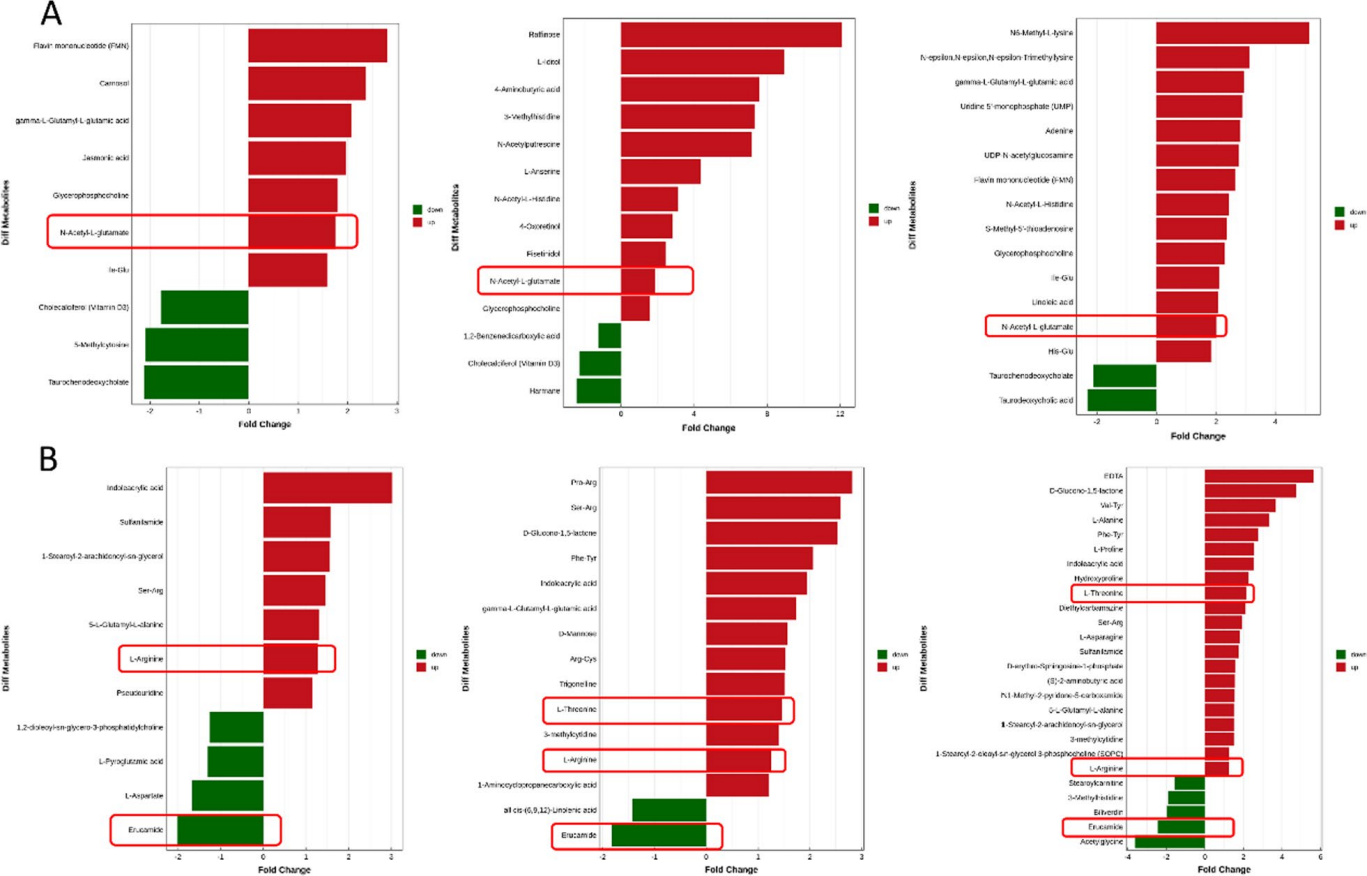
Fold-change analysis of the different metabolites explained that the stool (**A**) and serum (**B**) of FMT treated patients were different from baseline

**Fig. 9:**
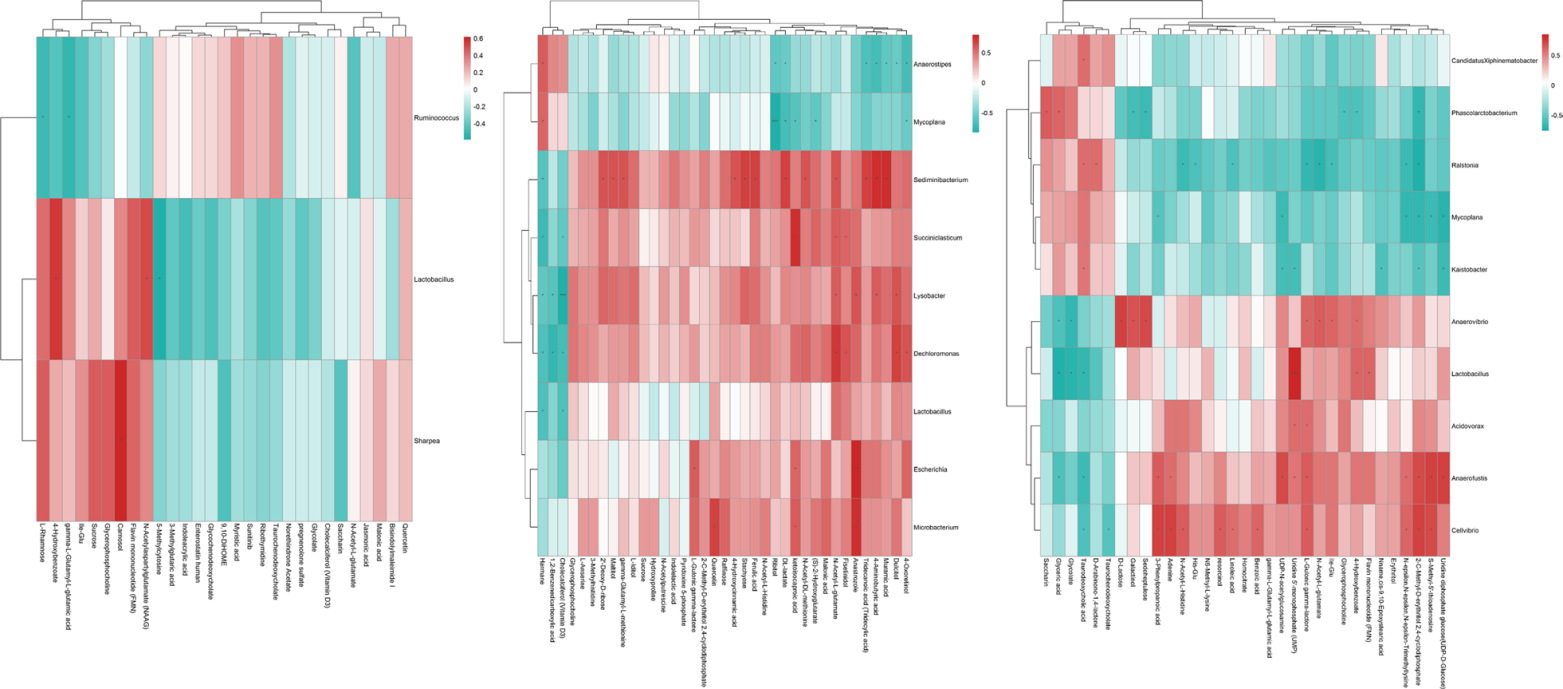
The correlation coefficient matrix heat map was used to show the associations between the metabolites and gut microbiota

**Fig. 10:**
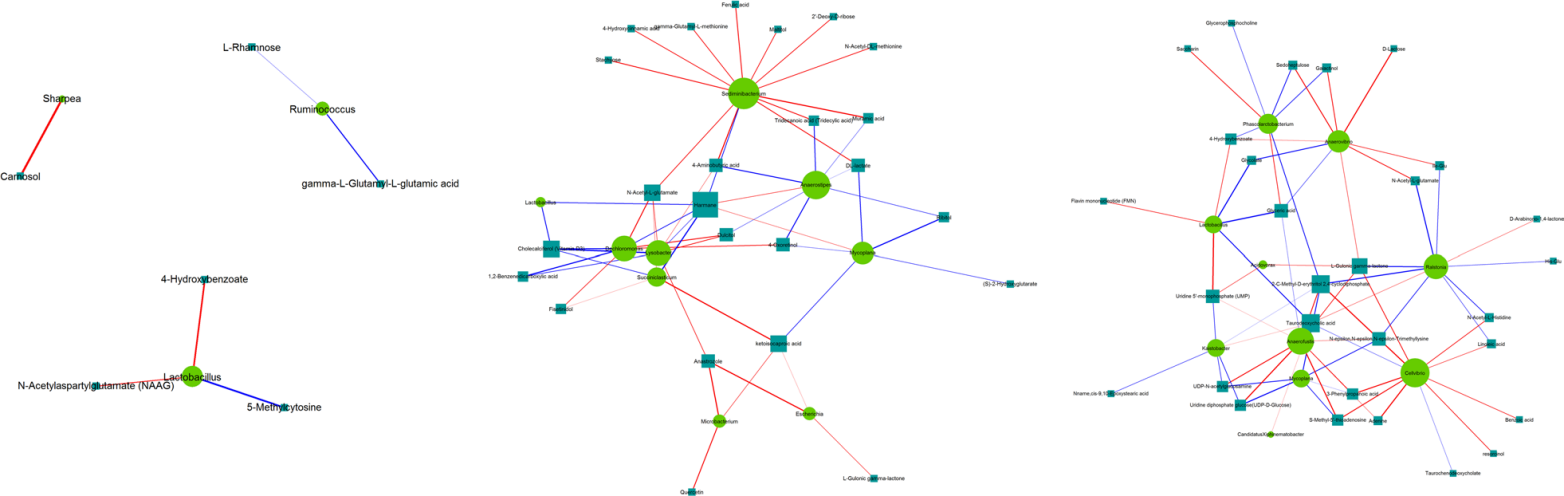
Network Diagram was used to show the associations between the metabolites and gut microbiota

**Fig. 11:**
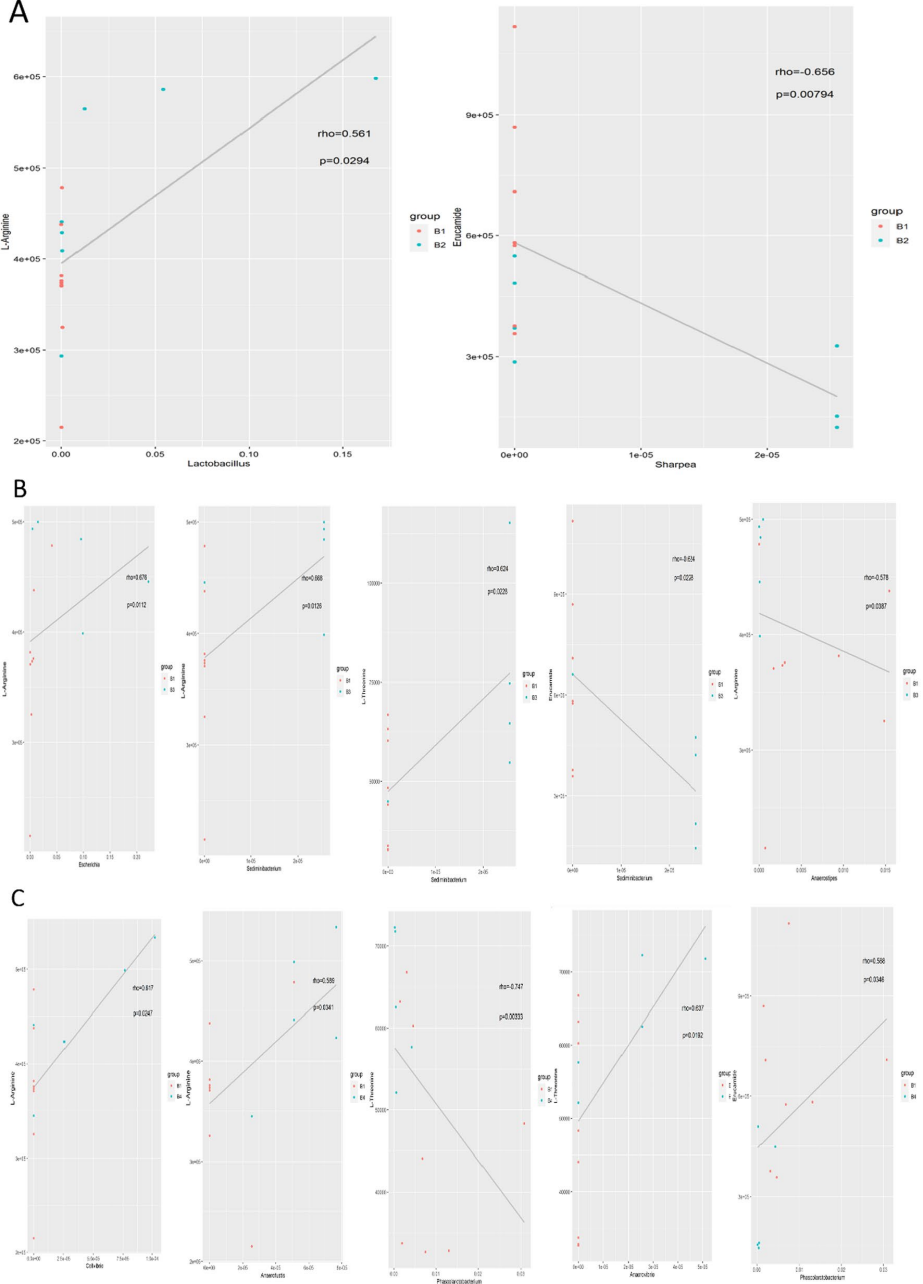
Scatter Plot was used to show the associations between the metabolites and gut microbiota

The metabolites produced by intestinal flora could significantly influence host physiology. Metabolomic analysis was used to assess the differential levels of metabolites in stools and serum of patients following FMT. We found that the metabolomic profiles of patients shifted after the FMT procedure. The orthogonal projections to Least Squares Discrimination Analysis (OPLS-DA) score plot, Volcano plot, Metabolites heatmap, Fold-change analysis of the different metabolites expressed the separation of clusters between the patients of baseline and post FMT. The multivariate statistical analysis of OPLS-DA showed that the cluster of metabolites was significantly separated between baseline and post-FMT, indicating that the metabolism both in feces and serum were changed after FMT (Fig. 5). The univariate statistical analysis such as Volcano plot and Metabolites heatmap also showed the metabolite changes between baseline and post-FMT (Figs. 6, 7). Fold-change analysis of the different metabolites explained that the stool of FMT treated patients were characterized by relatively high levels of N-Acetyl-L-glutamate, gamma-L-Glutamyl-L-glutamic acid, Glycerophosphocholine, et al. (Fig. 8A). At the same time, the serum of treated patients was characterized by relatively high levels of L‐Arginine, L‐Threonine, Ser-Arg, Indoleacrylic acid, Phe-Tyr, 5-L‐Glutamyl-L‐alanine, and lower levels of Erucamide, et al. (Fig. 8B). It was interesting that the N-Acetyl-L‐glutamate, which increased in feces, was involved in the L-Arginine biosynthesis according to the KEGG. Correlation analysis was conducted to evaluate the associations between the metabolites and gut microbiota. The correlation coefficient matrix heat map, Network Diagram and Scatter Plot provide the visualization of the relationship (Figs. 9, 10, 11). For example, L‐Arginine was positively correlated with lactobacillus (r = 0.561), Escherichia (r = 0.668), Sediminibacterium (r = 0.624), Cellxibrio (r = 0.617), and Anaerofustis (r = 0.559), while negatively correlated with Anaerostipes (r = − 0.578). L-Threonine was positively correlated with Anaerovibrio (r = 0.637), Sediminibacterium (r = 0.624) and negatively correlated with Phascolarctobacterium (r = − 0.747). Erucamide had significant negative correlations with Sediminibacterium (r = − 0.624) and Sharpea (r = -0.656), while being significantly positively correlated with Phascolarctobacterium (r = 0.588).

### L‐Arginine and L-Threonine are involved in the protein digestion and absorption pathway

**Fig. 12:**
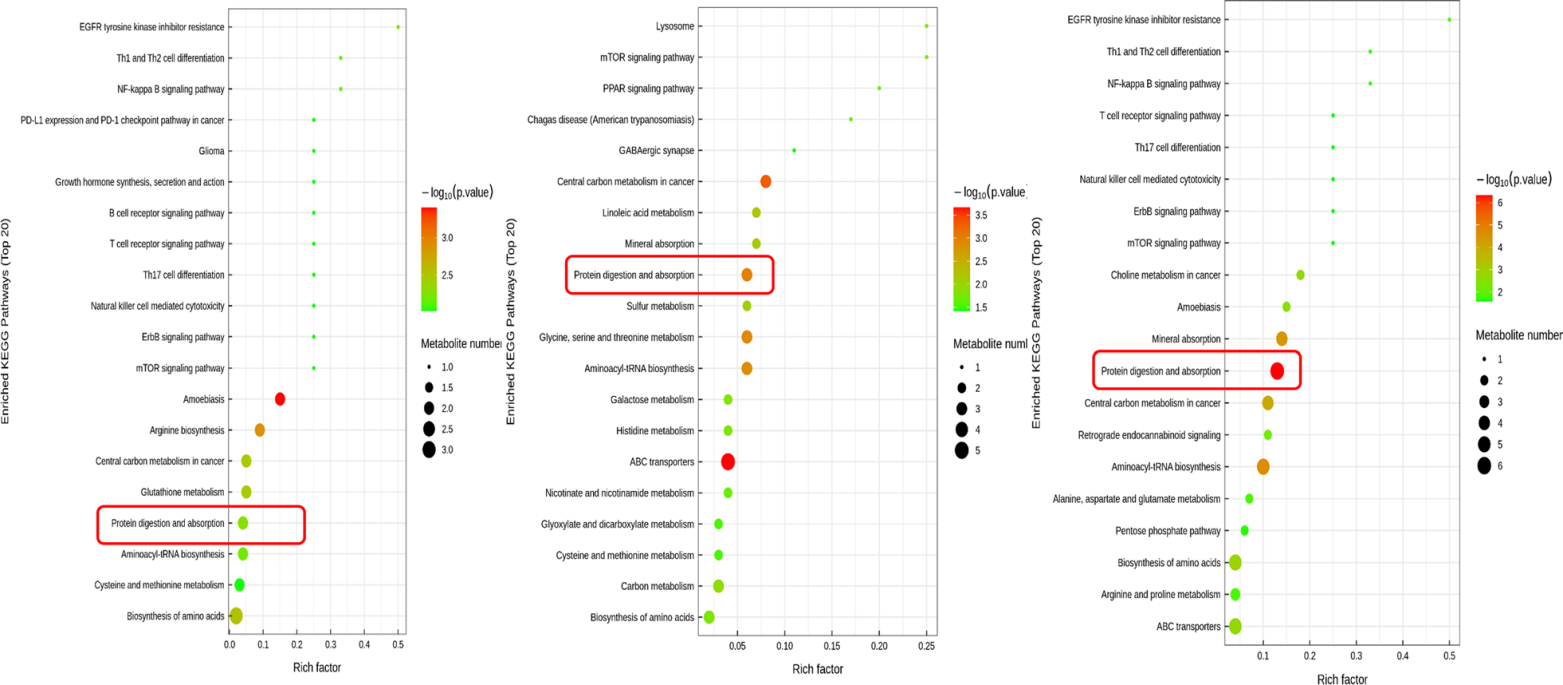
KEGG analysis was used to further investigate the mechanism of intestinal flora and metabolites in the treatment of STC. The protein digestion and absorption pathways gradually upregulated with the increase of FMT frequency

**Fig. 13:**
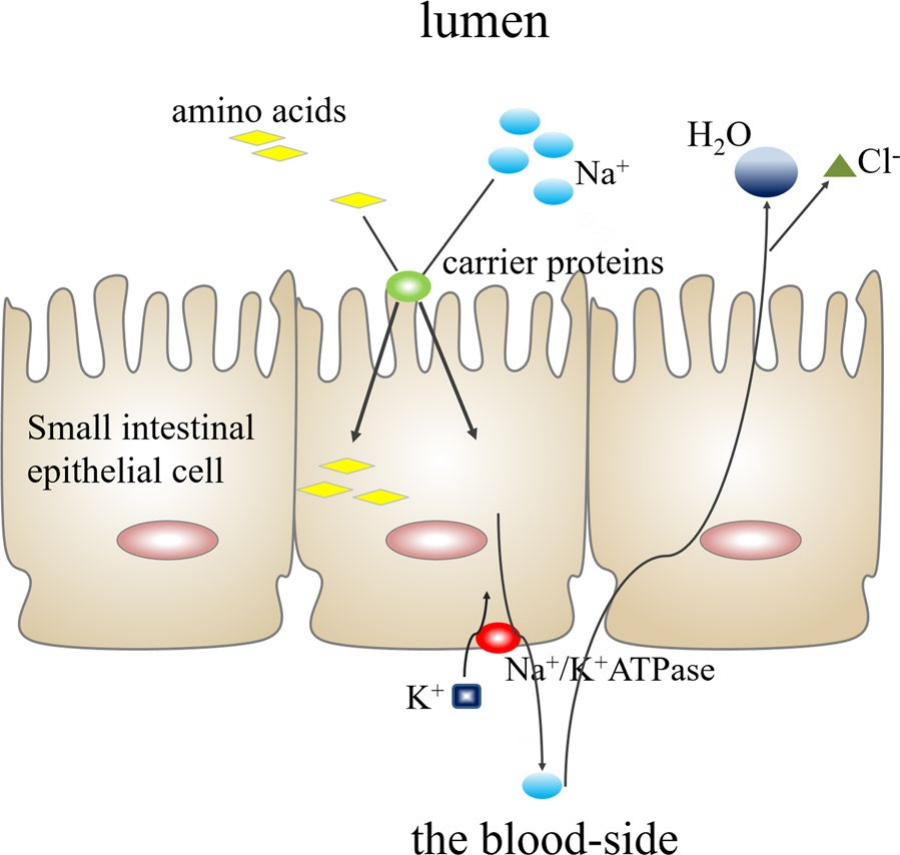
The protein digestion and absorption pathways mapper

To further investigate the mechanism of intestinal flora and metabolites in the treatment of STC, we conducted the KEGG analysis (Fig. 12). Enriched KEGG pathways analysis demonstrated the significant pathways involving metabolites. We noticed that the protein digestion and absorption pathways gradually upregulated with the increase of FMT frequency. At the same time, the L‐Arginine and L-Threonine were involved in the pathway. The protein digestion and absorption pathways were further shown to explore the function through the pathway mapper (Fig. 13). The exogenous protein and endogenous protein were digested into amino acids and oligopeptides by pepsin and pancreatic enzyme. Many carrier proteins transport amino acids and oligopeptides into small intestinal epithelial cells. The carrier protein can combine amino acids and Na + to transport amino acids into the cells, and Na + is removed from the cells with the help of sodium pumps, accompanied by the consumption of ATP. The protein that had not been digested and absorbed produced short-chain fatty acids under the putrefaction of the intestinal flora. We observed that a lot of Na + is absorbed from this pathway. The research showed that electrolyte and colonic fluid handling were the important treatments for constipation [1]. Transport of fluid and electrolytes in the intestine is critical for lubrication in order for the contents of the intestine to be propelled along its length [24].

## Discussion

A large number of studies have shown that disorders of the intestinal flora can cause STC, and that FMT has an effect on STC. In the present study, the clinical improvement rate was 62.5% (5/8), clinical remission rate was 75% (6/8), and the WCS significantly decreased in patients after the third FMT treatment. The treatment also improved the patient’s quality and relieved the depressive symptoms of patients based on the GIQLI scores and HAMD. On the whole, FMT not only improves the symptoms of constipation, but also relieves depression. Patients who received FMT treatment had several adverse events, too.

Then, we collected the feces and serum from the patients of baseline and post FMT to characterize the differences of microbiota and metabolite based on 16S rRNA sequencing and metabolomics. Compared to previous studies, we found that the ecological diversity and richness in the fecal microbiome of constipated patients were different from post-FMT, indicating that constipation was significantly associated with an altered gut microbiome. As a whole, our results showed that the fecal microbiome of constipated patients exhibited an increased level of Bacteroidetes and decreased level of Proteobacteria/Actinobacteria from the Phylum. Lactobacillus, Bifidobacterium/Bacteroides, et al., decreased on the level Genus. After treatment, the numbers of these flora reversed. Together, these results showed that positive alterations of microbial community richness and diversity were exerted by FMT, suggesting that this might be a potential therapeutic mechanism [25]. The gut microbiota in patients with constipation are different from the healthy controls, manifesting as the increasing abundance of Bacteroides and the decreasing abundance of Proteobacteria [26]. Khalif et al. found that Bifidobacteria and Lactobacillus significantly decreased in the 57 adult patients with functional constipation; this result is consistent with our study [27]. Moreover, it has been reported that Lactobacillus and Bifidobacterium can be used to prevent and relieve constipation [28–30]. Colonization with the specific microbiota such as Lacidophilus and Bifidobacterium bifidum in pathogen-free rats also normalizes small-bowel migrating motor complexes and gut transit time [31]. Fecal qRT-PCR showed a significant low value in Bifidobacterium and Bacteroides species in fecal specimens from constipated patients when compared to healthy controls [32]. There are many pathological damages in the intestinal epithelial cells of constipated rats: the depth of colonic crypt in constipated rats decreased, goblet cells were lost, et al. [33]. However, Lactobacillus can enhance intestinal epithelial barrier function and promote intestinal growth. Lactobacillus paracasei can increase short-chain fatty acid levels and promote intestinal peristalsis to relieve constipation [34, 35]. Meanwhile, Lactobacillus can promote intestinal electrolyte absorption by stimulating butyrate absorption and increasing Cl− /HCO_3_− and Na+ /H+ transport [36, 37].

The metabolomic analysis showed the metabolite both in stool and serum changed significantly after FMT. The present study found that N-Acetyl-L-glutamate, gamma-L-Glutamyl-L-glutamic acid, and Glycerophosphocholine were significantly increased in stool after treatment. L-Arginine, L-Threonine, Ser-Arg, Indoleacrylic acid, Phe-Tyr, 5-L-Glutamyl-L-alanine levels were significantly increased and the level of Erucamide decreased in serum compared to the baseline. Especially, we found that the N-Acetyl-L-glutamate detected in feces is involved in the synthesis of L-Arginine. The research has shown that L‐Arginine significantly inhibited the contraction in the distal colon to relieve symptoms of constipation and that L-Threonine is also associated with gastrointestinal diseases [38, 39]. It has been elaborated that nitric oxide(NO) plays an important role in relaxation of the smooth muscle in the gastrointestinal tract and the reduced production of NO may be an important contributing factor to gastrointestinal motility dysfunction [40]. L-Arginine and nitric oxide synthases (NOS) can synthesize NO to relieve symptoms of constipation [41–43]. L-Threonine is involved in the metabolism of vitamin B6, which is involved in the formation of the neurotransmitter serotonin (5-HT) [39]. The 5-HT regulates the central nervous system and enteric nervous system (ENS), including gastrointestinal (GI) motility and mood. Meanwhile, a deficiency of 5-HT can cause brain and intestinal dysfunction. Administration of slow-release 5-HTP to mice model of depression reduced depressive-like behaviors and promoted the growth of intestinal epithelium to normalize GI transit [44]. Indoleacrylic acid is one of the metabolites of tryptophan, and tryptophan catabolites are important contributors to intestinal homeostasiss [45]. In the antidepressant experiment, Erucamide induces the immobility time in the forced swimming test and tail suspension test which are used to evaluate depressive-like behavior. So Erucamide regulates the central nervous system to antagonize depression [46]. Erucamide also can improve memory deficit [47]. Our research showed that the patients’ depression symptoms were reduced, and this may be related to Erucamide. At the same time, changes of these metabolites are related to the intestinal flora. For example, L‐Arginine was positively correlated with lactobacillus. Erucamide had significant negative correlations with Sediminibacterium and Sharpea, while being positively correlated with Phascolarctobacterium. The N-Acetyl-L-glutamate gradually had significant positive correlations with lactobacillus.

Further research shows that L‐Arginine and L-Threonine are involved in the protein digestion and absorption pathways which gradually upregulated in the KEGG pathways analysis. Intestinal epithelial cells absorb large amounts of Na + from this pathway. The imbalance of ion secretory and ion absorptive process in intestinal electrolyte transport can result in constipation and all of this is dependent on the Na + /K + ATPase. The main mechanism of mucus secretion in the intestine is the movement of Cl− from the blood-side to the lumen through epithelial cells, the subsequent electrical gradient drives the passive movement of Na + , and the osmotic gradient causes the movement of water into the lumen [48]. The liquid extraction in preparation of stool depends on electrogentic Na + absorption in the distal colon [49]. Sufficient small intestinal fluid secreted into the colon can change the consistency of stool and accelerate colonic transit [50, 51]. Our research shows that the protein digestion and absorption pathway produces more Na + in the intestine and may stimulate the intestinal mucosal epithelium secreting more water to relieve constipation. The motility of the GI tract is inseparable from electrical excitation and excitation–contraction coupling. Ion channels, which are the diverse group of pore-forming proteins, participate in the electrical signals of most tissues and, thus, generate every movement and perception [52]. Ion channels, particularly Na + channels, are the essential players in electrical excitability of GI smooth muscle; consequently, drug developers considered that voltage-gated ion channels could be used as molecular targets in GI motility disorders [53]. The Na^+^ channel Na_V_1.5, which was encoded by the *SCN5A* mRNA, decreased in the STC samples. A novel miRNA regulator of NaV1.5, let-7f, one of the overexpressed miRNA in STC, significantly decreased Na + current density and reduced motility of human smooth muscle. At the same time, overexpression of let-7f produced reduced GI smooth muscle contraction in animal experimentation [54].

A large amount of SCFA is produced in the process of protein digestion and absorption. SCFA is critical for colonic function, regulating colonic growth and differentiation, motility, blood flow, barrier integrity, and is a major source of metabolic fuel in the colon [48]. Furthermore, in the colon, the microbial metabolism of undigested carbohydrates produces SCFA which promotes fluid absorption by Na + and SCFA− transporters and by increasing colonic NHE3 and NHE8 activity and expression [48].

Therefore, FMT can improve the symptoms of STC and the possible mechanism is increasing the production of L-Arginine and other metabolites by intestinal probiotics. These metabolites participate in the protein digestion and absorption pathways to produce Na + , thereby alleviating STC.

## Data Availability

All the data are included in the article.
